# Harnessing personal and social resources in managing internalising and externalising symptoms in children living in low‐resource settings

**DOI:** 10.1002/jcv2.70113

**Published:** 2026-03-19

**Authors:** Julia E. Michalek, Kathryn Bates, Francois van Loggerenberg, Dennis Ougrin, Jennifer Y. F. Lau

**Affiliations:** ^1^ Youth Resilience Unit Centre for Psychiatry and Mental Health Wolfson Institute of Population Health Queen Mary University of London London UK; ^2^ Department of Psychology Institute of Psychiatry Psychology and Neuroscience King's College London London UK

**Keywords:** at‐risk children, low resource, mental health, resilience, resources

## Abstract

**Background:**

Children growing up in low‐resource settings are at greater risk for lifelong psychiatric problems. They are both more likely to have risk factors for early psychopathology and to be less likely to seek help and engage support for these problems. Resource‐oriented therapeutic models—those that emphasise strengths of individuals and harness positive personal and social resources—may be particularly crucial in reducing the lifelong health inequalities that could arise amongst children growing up in socially deprived areas.

**Methods:**

We used network analysis to investigate the use of personal (different emotion regulation (ER) strategies) and social (social connectedness, religion, recreational activities) resources of children living in East London (*N* = 867, *M*_age_ = 8.76 (0.95), 49% female) and their mental health outcomes using self‐ and teacher‐reported questionnaires (data collected November 2022–2023).

**Results:**

We found that family, peer, and school connectedness were the most central elements of the network, whilst negative ER strategies seemed to be a part of the cluster of anxiety and depression symptoms.

**Conclusion:**

Our findings highlight the importance of harnessing both internal strengths and positive social resources when thinking about intervention programmes for symptoms of emotional disorders in children growing up in deprived areas. Identifying strategies for nurturing social connectedness in children's closest environment is also crucial.

## Key Points

**What's known?**

- Children growing up in socially deprived settings are at heightened risk for life‐long psychiatric problems, and little is known about the different personal and social resources they draw on to manage their mental health difficulties.
- Resource‐oriented approaches emphasise the combined use of personal and social resources, but studies rarely investigate these two elements simultaneously.

**What's new?**

- Using a data‐driven network approach, we found that family, school, and peer connected were the most central resources used to manage mental distress in a cohort of east London pre‐adolescents.

**What's relevant?**

- Prevention and intervention efforts for emotional problems in children growing up in deprived areas should consider nurturing both children's personal strengths and their social connections.

## INTRODUCTION

The World Health Organisation estimated that in 2019, 970 million people globally were living with a mental disorder. The number of people reporting mental health difficulties has continued to rise since. Children and young people represent one age group that may have been especially affected by recent global stressors, including but not limited to the pandemic: a 2023 survey of 8–16‐year‐olds in the UK suggested that 20.5% had a probable disorder, whereas in 2017, this figure was 12.5% (Newlove‐Delgado et al., 2022; NHS England Digital, 2023). As these early‐onset conditions can affect broad areas of functioning, from family life to social relationships to educational attainments and employment opportunities—and affect mental and physical health outcomes in adulthood—urgent delivery of early interventions is a global health priority (McGorry et al., 2022). Yet, the current range of mental health services and frontline therapeutic interventions has not been adequate at providing support for this growing number of young people requiring treatment. This is especially true for families living in lower‐resource settings, who face the double disadvantage of being exposed to social determinants that amplify the risks for mental health difficulties and who are also less able to access front‐line treatments because of resource constraints (Kirkbride et al., 2024).

A resource‐oriented approach is one that encourages individuals to use their own personal and social resources to manage mental health difficulties (Priebe et al., 2014). As these assets are already present within an individual (they could be personal characteristics and traits) or in their social support networks and the wider community, they may be less reliant on trained and qualified professionals to deliver these. Thus, arguably, leveraging these in programmes could provide an alternative, and potentially cost‐effective approach, especially in contexts where formal channels of mental health support are limited (Sikira et al., 2022). Unlike deficit‐based models focusing primarily on symptom reduction, resource‐oriented approaches build on positive strengths and qualities, such as resilience. Contemporary developmental perspectives emphasise that resilience can include not only the capacity to bounce back from adversity and recover, but also the development of adaptive skills and prosocial competencies that arise in response to adversity. Such *adaptation‐* or *strength‐based* theories propose that growing up in harsh or unpredictable environments can foster specialised attention, learning, and social strategies that are adaptive in high‐stress and low‐resource contexts (Ellis et al., 2017; Frankenhuis et al., 2020). These emerging strength‐focused frameworks advocate for balancing deficit models with careful investigation of abilities and prosocial adaptations that may arise in context of scarcity or chronic stress (DeJoseph et al., 2024). Indeed, a low‐resource context may be associated with heightened social attunement and increased prosocial behaviour, indicating that such adaptations may function as assets rather than vulnerabilities under certain conditions (Piff & Robinson, 2017). Strength‐based perspectives align with the aims of the present study, highlighting the value of identifying which personal and social resources are most central to children's everyday lives. These resource‐based approaches might point to strengths that are more acceptable, less stigmatising, and potentially more sustainable as targets for prevention and intervention (DeJoseph et al., 2024; Priebe et al., 2014).

However, there is a gap in our understanding of how children and young people draw on different internal and external resources to manage mental health difficulties. Where studies have explored these, the focus tends to be on either internal traits or external factors, not both together. For example, quantitative and qualitative studies show the importance of different emotion regulation (ER) strategies and coping styles—with emerging findings suggesting that it is not just one or two strategies that are crucial, but rather it is the flexibility with which these strategies are used to navigate context‐specific challenges that is crucial in managing mental health struggles (Brandão et al., 2023; Brites et al., 2024). Other studies have focused on external factors—such as social support and connectedness with others in the family, school and neighbourhood, as well as sports, arts and other recreational activities (Kingsbury et al., 2020; Ozbay et al., 2007). However, this unifactorial approach to resource orientation ignores current socio‐ecological theories of resilience, which view this concept as a dynamic and multi‐factorial maturational process reflecting ‘the capacity of individuals to navigate their way to the psychological, social, cultural, and physical resources that sustain their well‐being, and their capacity individually and collectively to negotiate for these resources to be provided and experienced in culturally meaningful ways’ (Ungar, 2008, p. 225). This is also in line with well‐established developmental frameworks, such as Bronfenbrenner's bioecological model (Bronfenbrenner, 1977, 2000), which emphasises that children's development arises from nested and interacting systems: from individual (microsystem), to family and school (mesosystem), to broader societal contexts (macrosystems), highlighting the interplay between the child and their environment. The combinations of everyday systems including caregiver relationships, school environment, and self‐regulation skills are thusly crucial to the development of resilience and adaptive functioning (Masten, 2001). Taken together, these models emphasise how internal and social factors interact to protect children and young people from negative effects of adversity and support healthy development (Ungar et al., 2013; Vaughn & DeJonckheere, 2021). Indeed, themes emerging from earlier qualitative work in this population highlighted resilience as comprising both personal strengths and social support (Murray, Smith Scott, et al., 2025). A resource‐orientation approach should, therefore, view internal and external resources simultaneously.

### The present study

In this paper, we explore associations between children's reported use of a range of resources from internal ER strategies to external social and community resources. We use a network model to represent these different resources together to explore how they connect individually with symptoms but also how they co‐act and connect with one another. We conduct these analyses on data on internalising and externalising problems from a new cohort of primary school children, aged 7–11 years, growing up in east London. We focus on the pre‐adolescent age range because findings may more directly speak to preventative programmes around managing early emerging symptoms when they first onset but before they become stable and persistent. We focus on an east London cohort because children growing up in these boroughs may experience some of the highest levels of child poverty and associated social determinants of health (e.g., overcrowded housing, community violence, etc.) in the UK (Stone, 2023). For instance, the boroughs of Tower Hamlets and Newham rank one of the highest in England for children living in income‐deprived households, 71.3% and 59.7%, respectively (Ministry of Housing, Communities, & Local Government, 2025). Further, Trust for London reports that 47% of children in Tower Hamlets and 45% of children in Newham live in poverty, positioning it amongst the most deprived boroughs nationally (Trust for London, 2025). The characteristics of these neighbourhoods, including income deprivation, overcrowded housing, limited green spaces and other structural stressors, provide an important contextual setting for investigating resource use and mental health outcomes in youth.

In a series of pre‐registered analyses, we tested the following hypotheses: (1) *Firstly*, we expected the resources to cluster into two communities, with Cluster 1 comprising internal psychological resources, including ER strategies, and Cluster 2 comprising external resources in social networks, spirituality, and sports and recreational activities. Within the external resource cluster (Cluster 2), we hypothesised that social relationships will be the most central to the network. (2) *Secondly*, we expected a pattern of negative and positive associations between emotional regulation strategies and internalising symptoms: positive cognitive strategies will be linked to lower levels of emotional problems, and negative cognitive strategies, such as rumination, will relate to worse emotional problems. (3) *Finally*, we also hypothesised that higher levels of social connections, sports activities, and spirituality will be linked to reduced emotional and behavioural difficulties (internalising and externalising symptoms). We also set to investigate if the patterns of associations differ between genders; however, as this analysis is exploratory, we did not have any directional hypotheses. Finally, our aim was also to describe the rate of mental health outcomes in the Development of Emotional Resilience (DEER) cohort and explore these in relation to different demographic characteristics (sex/gender, age, ethnicity, SES proxy).

## METHODS

### Setting and participants

This project is part DEER observational cohort study investigating risk and resilience factors in 7–11‐year‐old children in east London. The current paper focuses on measures collected during the first wave of the study (November 2022–2023). Participants (*N* = 867, *M*_*age*_ = 8.76 years old, SD = 0.95, 49% female) were children attending one of 10 primary schools in east London. Most children (62%) were *Asian*/*Asian British*, and *White* (18%), followed by *Black/African/Caribbean/Black British* (9%), *Mixed/Multiple Ethnic Groups* (7%), and *Other ethnic group* (5%). 25% of children were receiving free school meals at (aka Pupil Premium) the time of data collection, which we used as a proxy measure of socioeconomic status (SES). However, we do not treat free school meals as a comprehensive indicator of SES, as its eligibility is determined by a narrow set of administrative criteria that is likely to underestimate the proportion of children experiencing material hardships. In England, free school meals eligibility during our data collection period required families to be in receipt of specific income‐related benefits, such as Universal Credit with net earned income not exceeding £7400 per year, or legacy income‐based support (Department for Education, 2018; Waters & Joyce, 2018). These households capture only the most economically disadvantaged household and exclude many families experiencing financial insecurity but not meeting the exact criteria, for example, households with fluctuating employment, immigration‐related barriers to claiming benefits, stigma, or income levels just above the threshold (Addis & Murphy, 2018; Campbell & Obolenskya, 2025; Gorard, 2012; Sahota et al., 2014). Accordingly, our proxy measure of SES should be interpreted with caution, as it only partially reflects socioeconomic variability in the sample.

### Ethical information

The study was granted ethical approval from the Queen Mary University Ethics Board (QMERC22.251). Children were recruited through participating east London schools in the boroughs of Newham, Redbridge, Waltham Forest, and Havering, with a mix of opt‐in and opt‐out procedures. Parents provided consent and children assent prior to taking part in the study.

Data were collected during school visits from groups of children across three testing sessions (1.5 h each). Children completed the surveys individually using Qualtrics on Android tablets, with research assistants present to guide participants and answer questions. Most questionnaire measures were self‐reported by the children, but demographic information regarding child ethnicity and socioeconomic status were reported by the schools. Child externalising symptoms were reported by their class teachers, however, staff shortages and busy schedules of teachers meant that questionnaires for only 504 children were returned.

### Mental health measures

*Revised Child Depression and Anxiety Scale* (RCADS, Chorpita et al., 2000) measures children's internalising problems, with the focus on symptoms of generalised anxiety (6 items) and depression (10 items), on a 4‐point Likert scale ranging from 0 to 3 (*Never‐ Always*). Generalised anxiety scale includes questions such as ‘*I worry that bad things will happen to me’*, and depression scale items like ‘*I feel sad or empty’*. Here, we calculated t‐scores using the developer’ syntax (accounting for child age and gender) for the generalised anxiety subscale (*α* = 0.63) and depression subscale (*α* = 0.77). The final t‐scores all range from 0 to 100, with higher scores indicative of more symptoms. We opted to use the self‐reported measures of anxiety and depression through the RCADS as internalising symptoms may be more accurately reported by children themselves (Caqueo‐Urízar et al., 2022).

*Strengths and Difficulties Questionnaire* (SDQ, Goodman et al., 1998) is the only teacher‐reported, paper‐based measure in the current study. SDQ assesses general emotional and behavioural difficulties across five domains but our analysis used only two domains concerned with externalising (behavioural) symptoms: conduct (5 items, *α* = 0.75), including items such as ‘*Often fights with other children’* and hyperactivity/inattention (5 items, *α* = 0.88), with items such as ‘*Easily distracted*, *concentration wanders’*. The scale is scored on a 3‐point Likert scale from *Not true* (0) to *Certainly true* (2). Positive items were reverse‐scored, and scores were calculated by summing all items within the subscale, with higher scores suggesting more externalising difficulties. SDQ scores were only available for a subset of children (*n* = 504).

### Resource measures

*Cognitive and ER Questionnaire short‐form* (CERQ‐k, Garnefski et al., 2007) contains 18 items and measures 9 (adaptive and maladaptive) ER strategies: self‐blame, other‐blame, acceptance (‘*resignation of what happened*’), planning, positive refocusing, rumination, positive reappraisal, putting into perspective, and catastrophising. Children are asked how often they use different strategies when something unpleasant happens, for example, ‘*I think that I can learn from it*’, on a five‐point Likert scale ranging from *(Almost) never* (1) to *(Almost) always* (5). The items in each subscale are summed for the total score of each ER strategy, with a higher score indicating more frequent use of each coping strategy. CERQ‐k showed good internal reliability in our sample (*α* = 0.77).

*Social Connectedness Questionnaire* (SCQ, Jose et al., 2012) is a measure of how socially connected the child is and how well they maintain these bonds. We only included the 28 items that measure connectedness (*α* = 0.91) in our study. These items span five domains reflecting 5 sub‐scales: family (e.g., ‘*My family asks each other for help*’), school (e.g., ‘*I like going to school*’), peer (e.g., ‘*I can trust my peers with personal problems*’), and community (e.g., ‘*My family and I can count on our neighbours for help*’) on a five‐point Likert scale ranging from *Never* (1) to *Always* (5). Items in each subscale are averaged, with higher scores suggestive of a more well‐connected social network.

*Youth Spirituality Scale* (YSS, Sifers et al., 2012) measures children's spirituality. Here, we used 10 items (*α* = 0.88), of the original 20, which tapped into the Relationship with God (8 items, e.g., ‘*How sure are you that there is a God*, *Higher Power*, *or Ultimate Reality?*’) and Religious Practices (2 items, e.g., ‘*How often do you want to worship* (*go to church*, *temple*, *synagogue*, *mosque*, *have ceremonies*)?’) subscales. Children rated each item on a five‐point Likert scale from *Not at all* (1) to *Very much* (5). Scores within each subscale are averaged, with higher scores indicating higher levels of spirituality.

*Children's Leisure Activities Study Survey* (CLASS, Telford et al., 2004) measures the average levels of physical and leisure activity reported by the children. It consists of 38 items asking children to report their usual levels of each activity in a typical week scored on a 4‐point Likert scale from *Not at all* (1) to *Three or more times a week* (4), resulting in two subscales: physical (sports) activities (28 items, e.g., football, skipping rope, *α* = 0.88) and leisure activities (10 items, e.g., reading, playing board games or cards, *α* = 0.64).

### Data analysis

All analyses were performed using R (R Core Team, 2024) in RStudio (Posit team 2025). First, we used the Mann‐Whitney *U* test to investigate potential internalising and externalising scores differences by sex and SES groups, the Kruskal‐Wallis test for differences by ethnicity, and linear regression for effects of age. We computed correlations using pairwise deletion to handle missing data.

We fit the network model of child‐reported resources: ER (9 variables), social connectedness (4 variables), spirituality (2 variables), sports and leisure activities (2 variables), child‐reported internalising symptoms (2 variables) and teacher‐reported externalising symptoms (2 variables). We adopted a network approach because it treats psychological constructs as a system of interacting components, in which nodes (symptoms, psychological resources and social resources) are joined by edges representing unique conditional associations after controlling for all other variables in the system (Borsboom et al., 2021). These explicit representations reveal how internal and external resources can co‐occur, co‐activate, and act as links between different clusters, allowing for identification of central and most influential within the network. This approach complements traditional latent/variable‐centred models by mapping potential pathways of cross‐level interactions between resources and outcomes.

We used the *bootnet* package (Epskamp et al., 2018) to estimate the two networks based on the Gaussian graphical model with the default tuning parameter of 0.5 (GGM, Costantini et al., 2015), employing GLASSO (graphical least absolute shrinkage and selection operator) regularisation (Tibshirani, 1996) which ensures the sparsity of the model (model with fewest edges) with the extended Bayesian information criterion (EBIC, Chen & Chen, 2008) to select the best fitting model. We used exploratory graph analysis (EGA, Hfgolino/EGA, 2021) with the Walktrap algorithm to identify nodes highly interconnected with other nodes in the network (clustering). To assess the overall structure of the network, we calculated several centrality indices. Of note, centrality refers to the extent to which a certain node has many connections to other nodes in a network and can influence or spread activation throughout the network. Different centrality indices provide insights into different dimensions of this influence. *Node strength* indicates how strongly and frequently a node is directly associated (has edges) with other nodes, based on the sum of the weighted number and strength of all connections of a specific node relative to all other nodes. *Node closeness* refers to the average distance (path) from a node to every other node in the network (shortest path between the nodes). It, therefore, considers *indirect* connections between nodes. A high closeness index indicates a short average distance of a specific node to all other nodes, which could reflect how quickly changes in that part of the network can affect changes in other parts of the network (Hevey, 2018). *Betweenness centrality* suggests how important nodes are to the flow of information in the network and the higher the betweenness, the more important the node is for efficient information flow across different nodes. *Expected influence (EI)* is a measure of node centrality that considers directionality (i.e., it distinguishes positive and negative edges) in the number of times a node is directly linked to other nodes (Robinaugh et al., 2016). Finally, we also used the correlation‐stability (CS) coefficient and the bootstrapped different test for edge‐weights and centrality indices to estimate the overall accuracy and stability of the network.

In our exploratory analysis, we estimated network models separately for boys and girls and used the *NetworkComparisonTest* package (van Borkulo et al., 2022) to compare the models for differences in network (possible edge weight differences) and global strength invariance (possible difference on the absolute sum of network edge weights). All analyses were preregistered on the OSF (https://osf.io/7a2qc).

As teacher‐reported data were only available for a subset of participating children, estimating the full network that included SDQ nodes required complete SDQ data. Therefore, we estimated our main network model on participants with complete SDQ data only (*n* = 504). To assess whether restricting the sample affected the network results for internalising symptoms and personal and social resources, we also estimated a network excluding the SDQ nodes on the full sample with all complete data available (*n* = 727). We further compared participants with and without SDQ data on key demographics and study variables and found no systematic differences (*p* > 0.05), suggesting that excluding those without SDQ data did not appear to change the sample composition. Results of additional analyses are reported in the Supplementary Materials, including resource‐only model details (Supporting Information S1: Appendix S1 and Figure S1), bridge strength details (Supporting Information S1: Appendix S2 and Figure S2) and networks with and without SDQ data (Supporting Information S1: Figure S3).

## RESULTS

### Children's internalising and externalising symptoms

Descriptive statistics are presented in Table 1. Boys and girls did not differ in their depression (*U* = 89,609, *p* = 0.13) or anxiety scores (*U* = 90,433, *p* = 0.08), but did vary on teacher‐reported externalising outcomes, with boys scoring higher than girls in conduct problems (*U* = 32,250, *p* < 0.001), and hyperactivity (*U* = 35,338, *p* < 0.001).

**TABLE 1:** Descriptive statistics for the total sample and disaggregated by sex.

	Total		Male		Female	
	*M* (SD)	*n*	*M* (SD)	*n*	*M* (SD)	*n*
Child reported						
Age	8.76 (0.95)	837	8.71 (0.95)	417	8.81 (0.94)	406
RCADS						
Anxiety	46.22 (11.64)	822	46.95 (12.13)	416	45.47 (11.09)	406
Depression	50.09 (11.38)		50.79 (11.96)		49.37 (10.72)	
CERQ‐k						
Self‐blame	4.20 (1.97)	805	4.17 (1.98)	398	4.21 (1.92)	390
Blaming others	3.71 (1.93)		3.83 (1.94)		3.56 (1.87)	
Acceptance	4.58 (2.13)		4.69 (2.15)		4.43 (2.09)	
Rumination	4.99 (2.15)		5.07 (2.15)		4.87 (2.13)	
Catastrophising	4.69 (2.20)		4.85 (2.25)		4.49 (2.15)	
Refocus on planning	6.87 (2.26)		7.11 (2.27)		6.65 (2.24)	
Positive refocussing	6.77 (2.41)		6.94 (2.39)		6.60 (2.42)	
Positive reappraisal	6.22 (2.30)		6.39 (2.30)		6.06 (2.29)	
Putting into perspective	5.56 (2.30)		5.67 (2.31)		5.45 (2.28)	
SCQ						
Family	45.16 (7.30)	801	45.28 (7.24)	395	45.29 (7.24)	389
School	23.91 (4.77)		23.38 (5.04)		24.54 (4.38)	
Peer	28.35 (5.09)		28.13 (5.09)		28.62 (5.10)	
Community	14.49 (3.33)		14.46 (3.39)		14.53 (3.26)	
CLASS						
Physical activity	55.27 (14.64)	731	54.84 (14.82)	358	55.81 (14.49)	358
Leisure activity	25.56 (5.59)	727	24.41 (5.65)	354	25.83 (5.42)	360
YSS						
Relationship with god	3.97 (0.85)	782	3.96 (0.88)	387	3.99 (0.82)	379
Religious practices	3.43 (1.20)		3.41 (1.21)		3.47 (1.17)	
Teacher‐reported						
SDQ						
Hyperactivity	2.97 (2.98)	504	3.65 (3.07)	241	2.20 (2.59)	228
Conduct problems	1.03 (1.66)		1.26 (1.79)		0.76 (1.40)	

Abbreviations: CERQ‐k = Cognitive and Emotion Regulation Questionnaire short form; CLASS = Child Leisure Activities Study Survey; *M =* mean; RCADS = Revised Child Anxiety and Depression Scale; SCQ = Social Connectedness Questionnaire; SD = standard deviation; SDQ = Strength and Difficulties Questionnaire; YSS = Youth Spirituality Scale.

Receiving free school meals did not affect child‐reported internalising symptoms (*U* = 55,414, *p* = 0.74 and *U* = 56,504, *p* = 0.45 for anxiety and depression, respectively), but teacher‐reported externalising symptoms did differ based on child's free school meals, with children receiving free school meals scoring higher than those who do not on both the hyperactivity (*U* = 18,739, *p* = 0.01) and conduct problems scale (*U* = 17,762, *p* < 0.001).

The symptoms of anxiety (*F*(1, 820) = 6.44, *p* = 0.01, *b* = −1.40, *AdjR* ^*2*^ = 0.01), depression (*F*(1, 820) = 12.25, *p* < 0.001, *b* = −1.46, *AdjR* ^*2*^ = 0.01) and teacher‐reported hyperactivity (*F*(1, 475) = 8.31, *p* = 0.004, *b* = −0.41, *AdjR* ^*2*^ = 0.02) all significantly decreased with age. Finally, ethnicity did not emerge as a significant factor in either self‐reported emotional (*F*(4, 764) = 0.71, *p* = 0.58 for anxiety; *F*(4, 125.78) = 0.29, *p* = 0.88 for depression) nor teacher‐reported externalising problems (*F*(4, 480) = 2.40, *p* = 0.05 for hyperactivity; *F*(4, 87.06) = 2.44, *p* = 0.05 for conduct). Demographic characteristics comparisons for all other variables are presented in Supporting Information S1: Table S1.

### Resources and mental health network model

**FIGURE 1:**
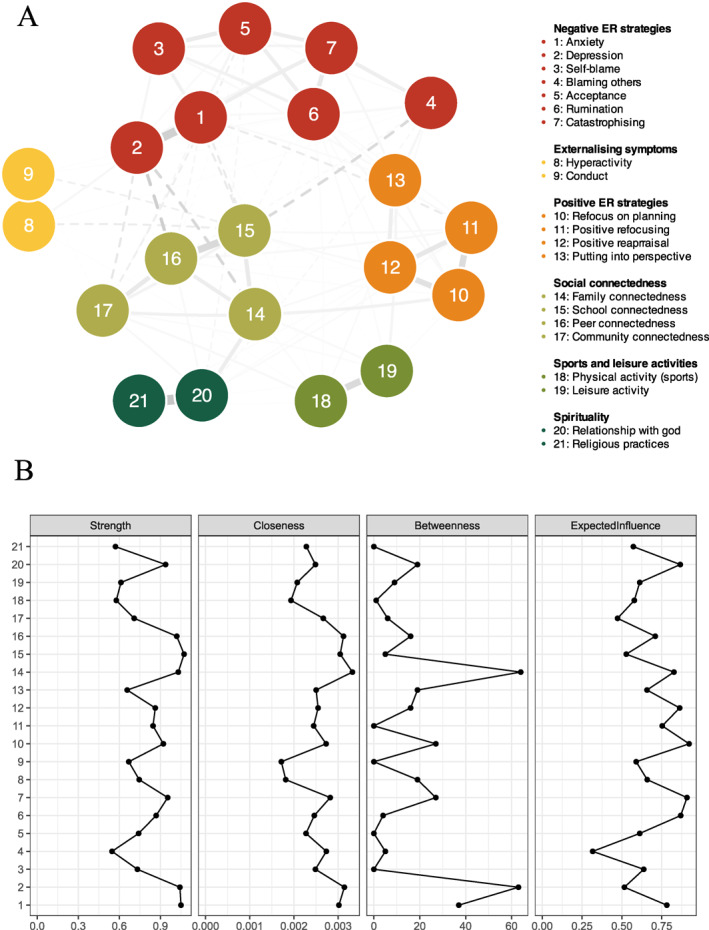
The main network model (A) and centrality indices (B). (A) Resources and mental health network model including 6 clusters. Smooth lines represent positive and dashed lines negative partial correlations, with line thickness indicative of the edge strength. (B) Centrality indices scores for each node within the network; *strength* indicates how strongly and frequently a node is directly linked to other nodes; *closeness* indicates the distance between different nodes and accounts for indirect connections; *betweenness* estimates how many shortest paths pass through a node; *expected influence* accounts for positive and negative connections.

In the network model of resources and mental health outcomes, the EGA identified six clusters across 21 nodes with 96 non‐zero edges and the mean weight of the network of 0.03, with edge weights ranging from −0.11 to 0.60 (Figure 1A). Cluster 1 included five negative ER strategies (self‐blame, blaming others, acceptance, rumination, and catastrophising) as well as child‐reported anxiety and depression symptoms. The teacher‐reported child problems with hyperactivity and conduct formed a separate cluster of externalising symptoms (Cluster 2). Cluster 3 contained four positive ER strategies (refocus on planning, positive refocusing, positive reappraisal, putting into perspective). Cluster 4 included the four social connectedness variables (family, school, peer, community). Cluster 5 contained two spirituality nodes (relationship with a God, religious practices) and Cluster 6 sports and leisure.

### Centrality indices and network accuracy

To assess the relationships between the resources and mental health, we estimated the node strength, closeness, betweenness, and EI (Figure 1B). Centrality estimates of the model were stable (>0.5), with *strength* and *edge* CS‐coefficients of 0.75, and *closeness* and *EI* CS of 0.52 (Supporting Information S1: Figure S4). *Betweenness* (estimating how frequently a node appears on the shortest path between other nodes) showed only acceptable stability (0.21), suggesting this centrality estimate should be interpreted with caution. Therefore, below, we report the centrality indices with the highest stability: node strength, closeness, and EI within the network.

*Node strength* indicates how strongly and frequently a node is directly associated (has edges) with other nodes. Anxiety (*strength* = 1.33) and depression (*strength* = 1.28) showed the highest node strength of the mental health problems cluster, with family (*strength* = 1.21), school (*strength* = 1.45) and peer connectedness (*strength* = 1.15) showing the highest node strength of the resources clusters, suggesting that these nodes are the most central to the network. These results indicate that internalising problems and school connectedness are the most strongly and frequently directly connected with other variables in the model.

*Node closeness* is the measure of distance between the different nodes and therefore also accounts for *indirect* connections. Family connectedness has the highest closeness index of the resources clusters and within the network (*closeness* = 1.74), followed by depression (*closeness* = 1.32) which has the highest closeness of mental health clusters. This suggests that family connections and symptoms of depression are the most *indirectly* connected to other outcomes, and both quickly *affect* and *are affected* by changes in other parts of the network (e.g., improvements in family environment are more likely to affect mental health outcomes than other types of resources in the model).

*Node EI* estimates the frequency and strength of connections with other variables considering whether these connections are positive or negative, that is, EI index will be high for nodes with many positive regularised partial correlations, and low for nodes with many negative correlations. Refocus on planning (*EI* = 1.51) and catastrophising (*EI* = 1.43) showed the highest EI in the model. This is driven by associations between refocus on planning and other adaptive ER strategies and social resources, and between catastrophising and other maladaptive ER strategies and internalising problems. The most negatively connected variable was blaming others (*EI* = −2.24), highlighting its strong negative association with school connectedness.

### Internalising symptoms and emotion regulation strategies

We hypothesised that emotional problems will be negatively linked to adaptive ER strategies and positively linked to maladaptive ones. Anxiety and depression symptoms were integrated into Cluster 1 (maladaptive ER strategies), with anxiety symptoms most strongly linked to catastrophising (*w* = 0.16) and depression symptoms associated with self‐blame (*w* = 0.13), suggesting strong links between negative coping strategies and emotional problems. Of note, depression was most strongly negatively linked to peer connectedness (*w* = −0.11), but the expected negative associations between adaptive ER nodes and internalising symptoms were weak or non‐existent in our model.

### Similarities and differences between boys and girls

To explore gender differences, we ran separate networks for boys (*n* = 241) and girls (*n* = 228). Both models included 21 nodes with mean network weight of 0.03. Centrality indices of *edge* and *strength* were stable (>0.52) but other indices showed moderate or poor stability. The EGA cluster detection identified 5 clusters in the *girls* network combining Clusters 5 and 6 (Spirituality with Sport and leisure) with 86 non‐zero edges. The *boys* network clusters remained the same as the full model across 73 non‐zero edges (Supporting Information S1: Figure S5). In the boys' network social connectedness was the most influential (highest node strength), whereas for girls this was replaced by adaptive and maladaptive ER strategies (internal resources). However, despite the descriptive characteristics on the two networks showing some variability suggesting subtle variation in node influence and clustering, the network comparison tests of network invariance (*M* = 0.12, *p* = 0.86) and global strength invariance (*S =* 0.58, *p* = 0.63) revealed that the two networks are structurally highly similar.

## DISCUSSION

This study investigated the use of personal and social resources amongst primary school‐aged children in East London. We used a data‐driven network approach to identify the central items and the pattern of communities in a network of internalising and externalising symptoms, personal (internal) resources like ER strategies and social (external) resources. Specifically, we aimed to test three hypotheses. Our first hypothesis concerned whether resources clustered into two communities, with one corresponding to internal psychological resources and the second comprising external resources. Our network model suggested six separate clusters. The largest cluster contained internalising problems but also included negative ER strategies, suggesting close relationships between internalising problems and maladaptive coping styles, like catastrophising and rumination. The second cluster described externalising problems (hyperactivity/inattention and conduct problems), with the remaining four clusters including positive cognitive‐regulation strategies (internal), social connectedness, sports and leisure activities, and spirituality. We expected that social relationships, that is connectedness with family, peers, school, and community, would be influential within the sub‐network of external resources, which was supported by our findings. Our second set of hypotheses related to negative and positive associations between emotional regulation strategies and internalising symptoms. Whilst negative ER strategies indeed were intricately linked with (worse) symptoms by appearing within the same cluster, the (independent) cluster of positive cognitive strategies was negatively linked to lower levels of emotional problems. Finally, we also found that higher levels of social connections, sports activities, and spirituality were linked to reduced internalising and externalising symptoms. Each of these findings is explored in detail.

Perhaps the most striking findings related to the co‐location of maladaptive cognitive styles and symptoms of anxiety and depression in the same cluster. Difficulties with accurately and appropriately regulating emotions have been widely shown to contribute to poorer mental health, with psychological models often positing these cognitive‐emotional factors as maintenance factors that become intertwined with symptoms in vicious cycles (e.g., Cisler & Olatunji, 2012; Lemoult & Gotlib, 2018). That is, rumination may prolong depressive mood, but the presence of low mood in turn increases ruminative tendencies (Joormann & Vanderlind, 2014). Our findings that these factors and symptoms were located in the same cluster may highlight this close (negative) synchrony. In contrast, adaptive and positive ER strategies, such as cognitive reappraisal, putting things into perspective, key protective factors instrumental in the development of resilience (Braet et al., 2014; Schäfer et al., 2017; Ursu & Măirean, 2022; Werner & Gross, 2010) were a separate cluster and less connected with these emotional symptoms. The ability to use adaptive ER strategies and coping with difficult emotions improves with age, and it may be that these personal internal resources will become more utilised (Sanchis‐Sanchis et al., 2020; Zeman et al., 2006) in a way that protects against anxiety and depression and instead predicts resilience. The transitional period from childhood to adolescence reflecting profound developmental changes in ER abilities (Gross, 2013), may mark an ideal time for teaching positive ER skills in preventative interventions so that they become more closely intwined with a reduced range of internalising problems in youth. This is particularly important, as childhood emotion dysregulation has been suggested as a precursor of internalising symptoms in later life (Murray, Wright, et al., 2025). Consistent with this, interventions aiming to disrupt the self‐reinforcing loop of maladaptive cognitions and emotional problems by teaching children cognitive reappraisal skills or mindfulness in place of rumination show promising results (Feldhaus et al., 2020; Hilt et al., 2025).

The overall centrality of social connectedness within the network suggested that social relationships could be a resource that children draw on to manage mental health distress. Alternatively, as these are cross‐sectional data, it could be that those with greater emotional and behavioural difficulties have reduced social connections. Regardless, school, family, and peer connections are most influential, with school connectedness having the most *direct* relationship with emotional and behavioural difficulties whilst family connectedness the most *indirect* effects. This is partly in line with previous literature which mostly emphasises the importance of family cohesion and support (Lucia & Breslau, 2006; McArthur et al., 2023; Roos et al., 2021). But the finding that school connectedness seemed to be the most important resource within the social connectedness cluster and in the network more generally (highest strength centrality) suggest that school connectedness associates not only with all symptom types (internalising, externalising) but also may activate the use of other resources within the child's social circle (e.g., peer connectedness) and their inner strengths (e.g., positive refocus). As children transition into adolescence, their peer relationships and socialising in the school environment becomes more salient (e.g., Kiuru et al., 2019; Nelson et al., 2016) (but see T. Cheng et al., 2024). It is therefore not surprising that reduced school connectedness associates with more symptoms of anxiety and depression, substance use, academic achievement, and fewer prosocial behaviours in teenage years (Bond et al., 2007; Oldfield et al., 2016; Shochet et al., 2006). These effects also extend to students' mental health outcomes even in remote‐learning contexts (Perkins et al., 2021). Studies tend to show reciprocal associations between these variables (Lester et al., 2013) but nonetheless, whole‐school approaches that aim to increase connectivity amongst pupils may be a promising avenue for building resilience and promoting wellbeing (Ungar et al., 2014). Together, our findings highlight the practical value of strengthening school connectedness as a scalable and impactful route for supporting youth wellbeing. Whole‐school initiatives can be effective in increasing students' sense of belonging and improving mental health outcomes (e.g., Bond et al., 2004; Wasserman et al., 2015). Embedding such connectivity‐enhancing practices into everyday school systems may help activate other key resources in the network, such as supportive peer relationships and adaptive coping, offering a feasible pathway for prevention and early intervention.

Importantly, the overall centrality of social connectedness and the distinct clustering of adaptive ER resources resonate with strength‐based perspectives on resilience in contexts of socioeconomic disadvantage (Theron, 2016; Ungar et al., 2013; Vaughn & DeJonckheere, 2021). In line with the adaptation‐based frameworks, heightened prosocial attunement and reliance on social networks may be an adaptive response to harsh and low‐resource environments, fostering cooperation and mutual aid that preserve functioning in contexts of material scarcity (Ellis et al., 2017; Frankenhuis et al., 2020; Piff & Robinson, 2017). This may be especially salient in East London, where large proportions of children live in low‐income households and experience intersecting social, educational, and environmental challenges (Ministry of Housing, Communities and Local Government, 2025). In this context, our findings suggest that supportive relationships function as a core adaptive resource, enabling children to draw on internal and external strengths to adapt positively to structural adversity. This also further highlights the need for socially and community grounded interventions that strengthen family, school, and peer networks to promote resilience and wellbeing in socioeconomically disadvantaged contexts.

Finally, we also tentatively found subtle non‐significant differences in the patterns of internal and external resource clusters between boys and girls. These may hint at possible gender differences in the types of resources most influential in coping with distress such that boys rely on social connections and girls on internal ER, but such potential differences should be investigated further, as the two models did not significantly differ in overall edge weight or global strength.

### Limitations

Our findings should be considered in light of some limitations. First, it is important to note that ours is not a clinical sample. In fact, most children in the current cohort scored within the *Normal range* according to expected norms (NHS England Digital, 2023) for generalised anxiety (92%) and depressive symptoms (90%), and within *Close to average* (Meltzer et al., 2003) for conduct problems (86%) and hyperactivity (81%). Thusly, findings may not generalise to those with more severe problems. Nonetheless, whilst mental health outcomes did not differ by ethnicity, receiving free school meals was linked to higher scores on externalising problems, in line with the current literature suggesting that poverty and economic deprivation are significant risk factors for children's behavioural problems (e.g., Flouri & Midouhas, 2016; McCulloch et al., 2000; Schonberg & Shaw, 2007). Our data provide insights on resource use to manage mental health symptoms amongst children growing up in environments with very high poverty rates (Stone, 2023), although future work should investigate resource networks using a more consistent and reliable individual poverty measure, such as the Index of Multiple Deprivation.

Second, although the *strength* index that we use as the main metric in our network showed good stability (0.75), *closeness* and *EI* were only moderately stable, whilst *betweenness* showed a particularly poor stability (0.21), meaning these need to be interpreted with caution. We, therefore, focused on *strength* which means that other key features of the network might have been lost.

Third, although we used measures developed for or validated in middle childhood and preadolescence (Liu et al., 2016; Orgilés et al., 2018), and internal consistency across the sample overall was acceptable, it is possible that some younger participants may have found certain cognitively abstract items more challenging to interpret (particularly some aspects of the ER scales). This raises the possibility of age‐related measurement error. Additionally, as we did not formally test measurement invariance across sociodemographic groups, our observed associations, strength, and placement of nodes within the network, might reflect differences in how items are interpreted rather than the true differences in underlying constructs. Future work should explore age‐related differences in item comprehension and include multi‐group confirmatory factor analyses to establish configural, metric, and scalar invariance to ensure comparability of scores across developmental and demographic groups.

Further, as this was primarily an exploratory, data‐driven analysis based on partial correlations on cross‐sectional data, we are not able to infer causal links between the key elements of children's internal and external resource use and mental health. These factors should be investigated longitudinally to disentangle the association between a more supportive family environment and adaptive ER strategies, and the mechanisms behind this relationship in order to fine‐tune intervention efforts. Similarly, due to sample size constraints, we were also unable to estimate networks for different demographic and developmental subgroups (e.g., age, SES, ethnicity), as recommended thresholds for reliable network comparison exceed our available group sizes (Forbes et al., 2021). Therefore, we have focused on the full‐sample model but note subgroup network comparisons are an important avenue for future research.

Lastly, although most of our measures were child‐reported, we used teacher‐reported measures of child externalising problems, which might have constituted reporting/measurement bias. Indeed, conduct problems and hyperactivity scales emerged as a separate, comparatively isolated cluster with relatively weak links to the other elements in the network, which may have been a result of reported differences and the reduced sample size. Although child, parent, and teacher reports of the SDQ externalising subscale show good validity and reliability (Becker et al., 2004; A. Goodman et al., 2010), discrepancies between reporters have been noted in previous studies (S. Cheng et al., 2018; Lewis et al., 2015). Further work should therefore explore the consistency between child‐, parent‐, and teacher‐reports for a more comprehensive understanding of children's behavioural issues.

Taken together, our findings suggest a strong link between family, school, and peer connectedness and better mental health, and highlight the need for a resource‐oriented approach with a combination of personal and social resources when attempting to tackle children's mental health difficulties. Further longitudinal research should investigate causal relationships between family cohesion, adaptive ER strategies, school connections, and internalising problems to be able to identify and develop appropriate prevention and intervention programmes that could provide the strongest support for building the resilience of children living in low‐resource settings.

## AUTHOR CONTRIBUTIONS

**Julia E. Michalek**: Investigation; writing—original draft; formal analysis; methodology; visualization; conceptualization; project administration. **Kathryn Bates**: Writing—review and editing; methodology. **Francois van Loggerenberg**: Writing—review and editing; conceptualization. **Dennis Ougrin**: Conceptualization; funding acquisition; writing—review and editing. **Jennifer Y. F. Lau**: Conceptualization; funding acquisition; writing—review and editing; supervision.

## CONFLICT OF INTEREST STATEMENT

The authors declare no conflicts of interest.

## ETHICAL CONSIDERATIONS

The study was granted ethical approval from the Queen Mary University Ethics Board (QMERC22.251; 27/07/2022). Parents provided consent and children assent prior to taking part in the study.

## Supporting information

Supporting Information S1

## Data Availability

The data that support the findings of this study are available from the corresponding author upon reasonable request.
