# Supplementary material for: Harnessing personal and social resources in managing internalising and externalising symptoms in children living in low‐resource settings
Source: JCPP Adv. 2026 Mar 19:e70113. Online ahead of print. doi: 10.1002/jcv2.70113 (PMC13339443; doi:10.1002/jcv2.70113)
Supplement: Supplementary file 1 — Supporting Information S1 [file JCV2-9999-e70113-s001.docx]

**Harnessing personal and social resources in managing internalising and externalising symptoms in children living in low-resource settings**

**Supporting Information**

**Appendix S1. Resource-only model**

In addition to the main (full) model, we also conducted a ‘resource-only’ model, in which the EGA identified five clusters across 17 nodes with 71 non-zero edges and the mean edge weight of .04 (Figure SM2). Cluster 1 contained five negative emotion regulation strategies (self-blame, blaming others, acceptance, rumination, and catastrophising). Cluster 2 contained four positive emotion regulation strategies (refocus on planning, positive refocusing, positive reappraisal, putting into perspective). Cluster 3 included the four social connectedness variables (family, school, peer, community). Cluster 4 contained two spirituality variables (relationship with a God, religious practices) and Cluster 5 contained physical (sports) and leisure activity variables.

**Figure S1. Resource-only network model (A) and centrality indices (B).**


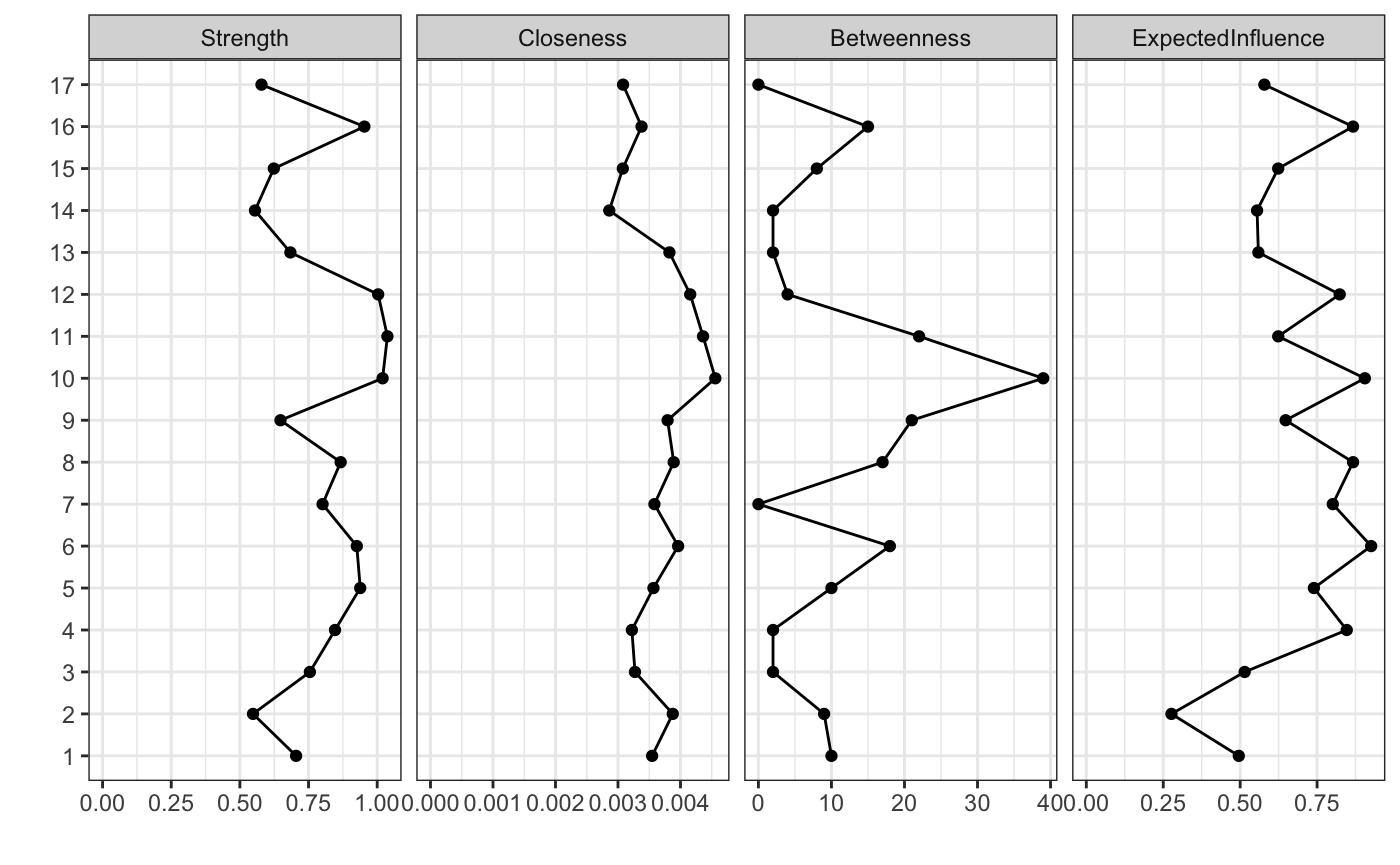

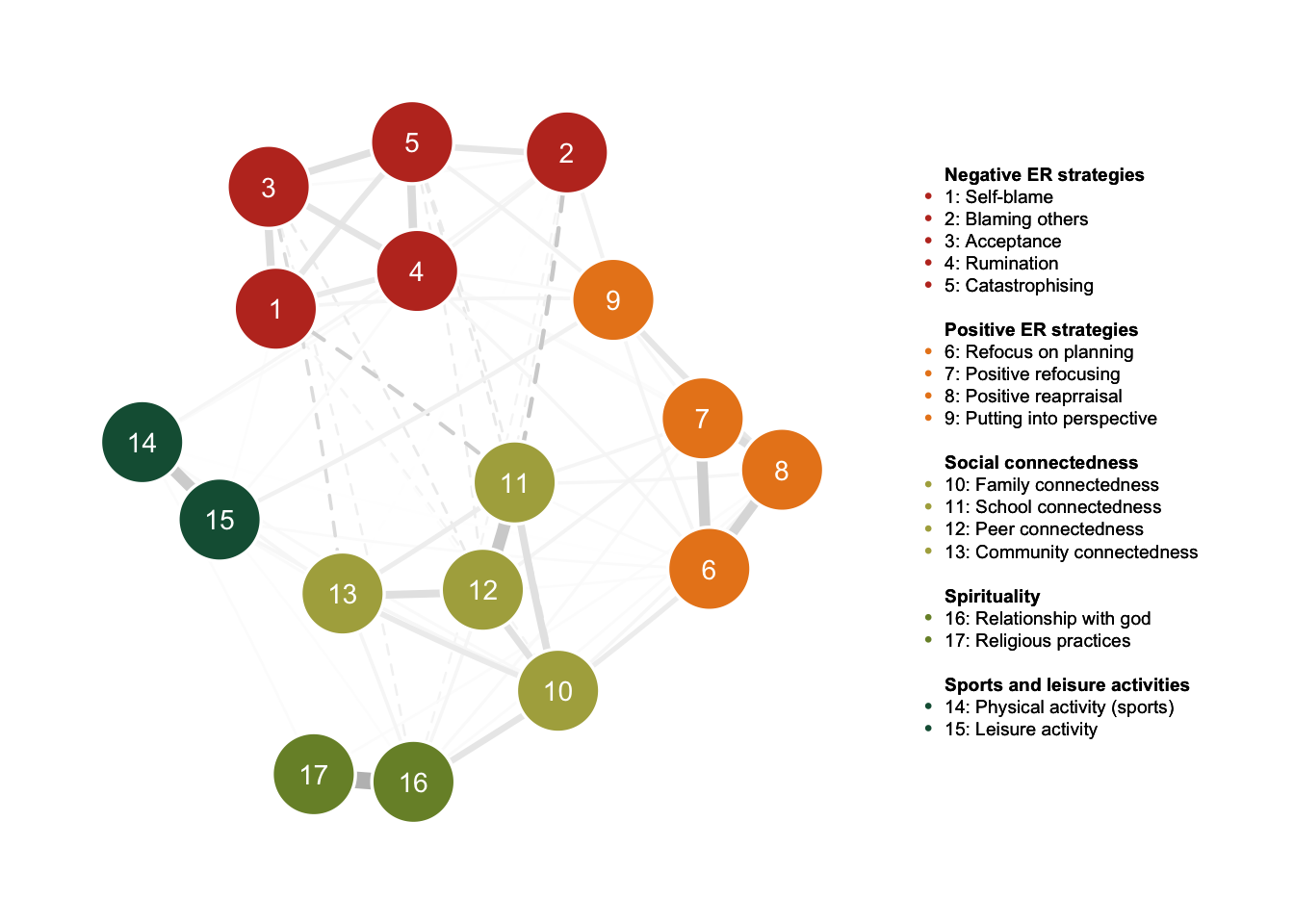


A

B

*Note.* A) Resources-only network model including 5 clusters. Smooth lines represent positive and dashed lines negative partial correlations, with line thickness indicative of the edge strength. B) Centrality indices scores for each node within the network.

**Appendix S2. Bridge strength**

To identify the key nodes within all resource clusters (i.e., nodes that function as ‘bridges’, connecting its cluster to other clusters in the network), we calculated *bridge strength* (Figure SM3B). Bridge nodes (nodes with the highest bridge strength) of the external resource clusters were *Family connectedness* (bridge strength = 0.54), *School connectedness* (0.42), and *Relationship with God* (0.41). *Putting things into perspective* showed the highest bridge strength in the internal resources cluster (0.38). Finally, *Depression* was the bridge node of mental health clusters (0.36).

As the node with the highest bridge strength in the model, *Family connectedness* was most strongly linked to nodes in other clusters of resources, namely *Refocus on planning* (*w* = .12) from Cluster 3, *Leisure activity* (*w* = .06) from Cluster 5, and *Relationship with god* (*w* = .17) from Cluster 6, suggesting that stronger family connections were linked to more recreational activities, higher levels of positive emotion regulation strategies and spirituality. In terms of mental health clusters, family connectedness was most strongly (and negatively) linked to depression (*w =* -.09), indicating that children who reported feeling more connected to their family also reported lower levels of depressive symptoms. Bridge nodes of the network are presented in Figure SM3A.

**Figure S2. Bridge nodes of the network and bridge centrality.**


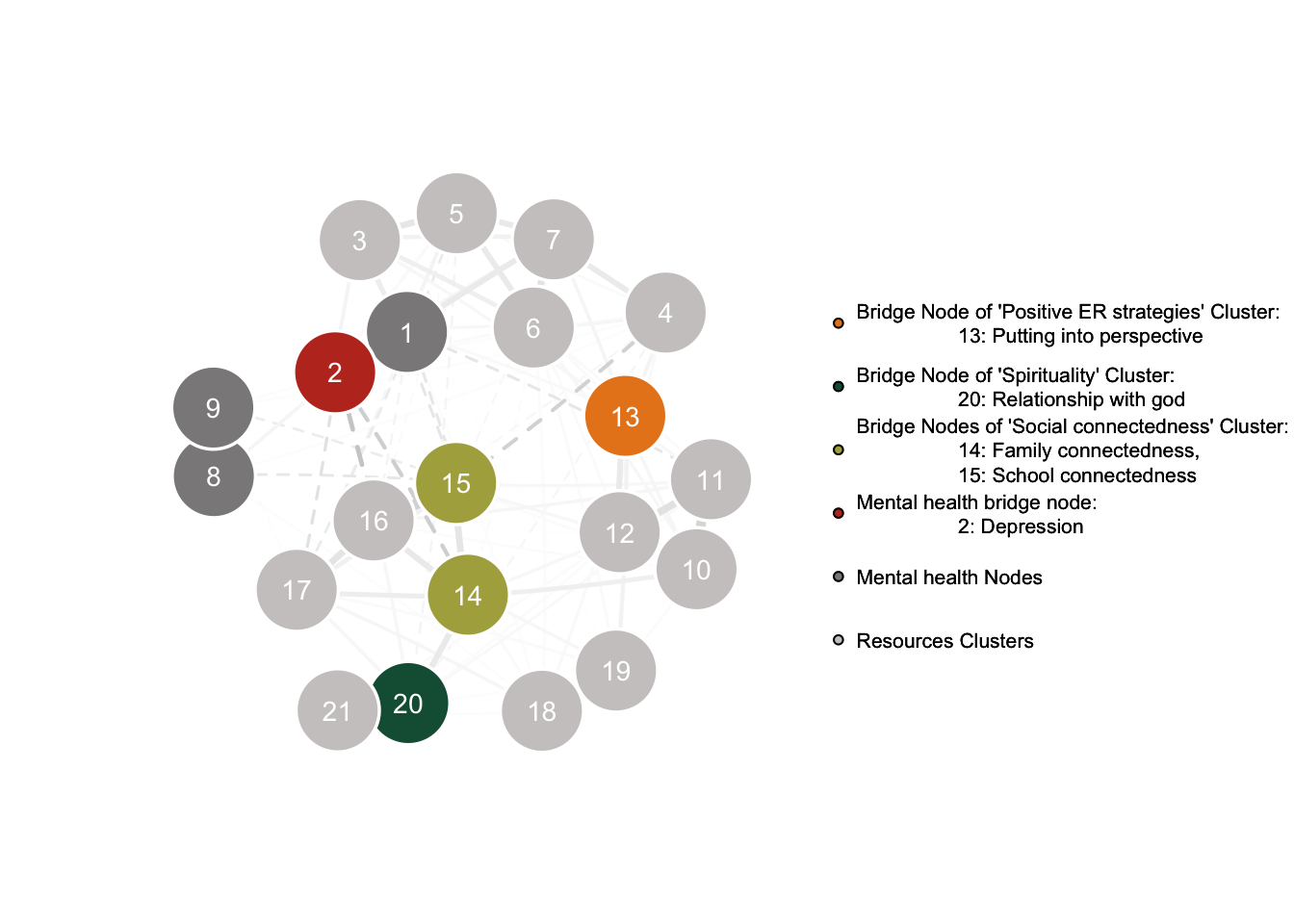


A

B


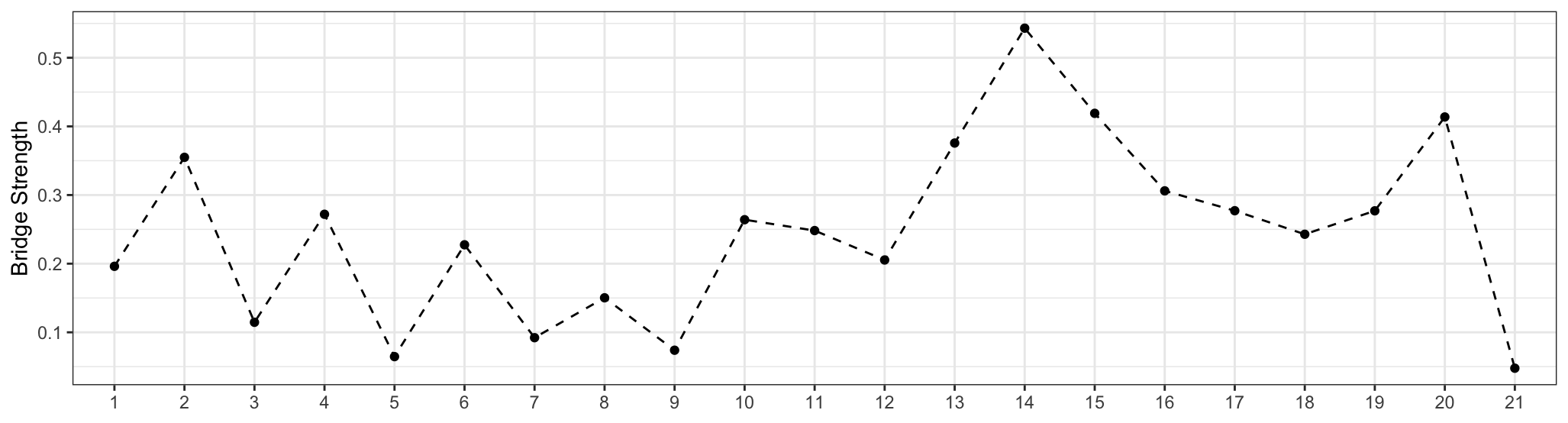


*Note*. A) Bridge nodes of the main network highlighted; B) Overall bridge strength index. X-axis numbered nodes as in Figure 1A in the main text.

**Figure S3. Network models with child-reported data only (*N* = 727).** 
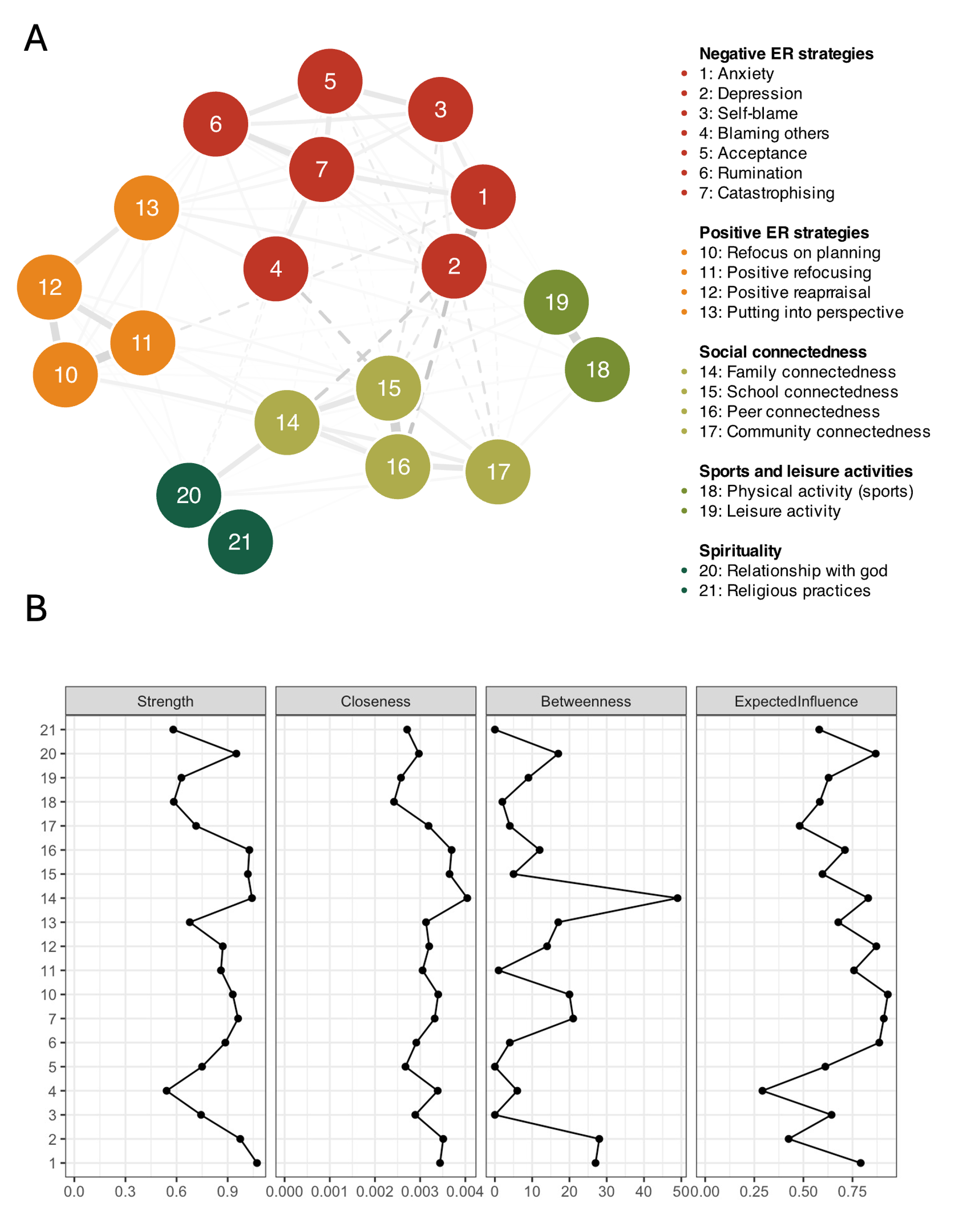


*Note.* A) Network models and node strength index of resources and internalising symptoms. B) Centrality indices scores for each node within the network. Here, anxiety (*strength* = 1.38), depression (*strength* = 0.81), and family (*strength* = 1.21), peer (*strength* = 1.12), and school connectedness (*strength* = 1.07) showed the highest node strength.

**
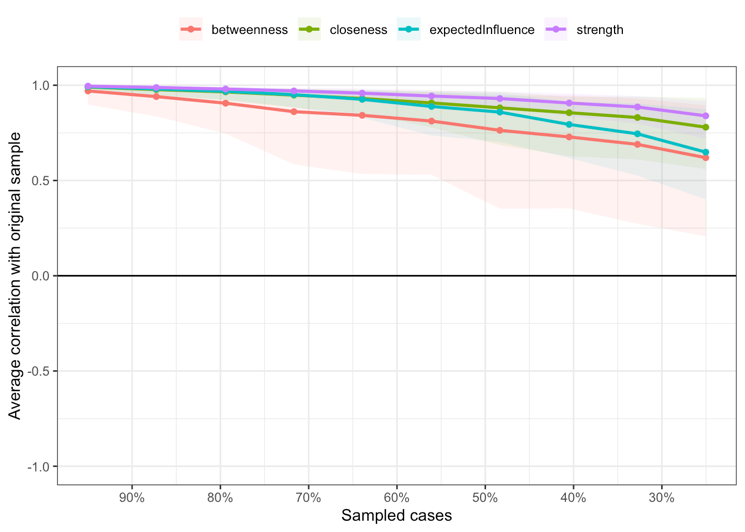

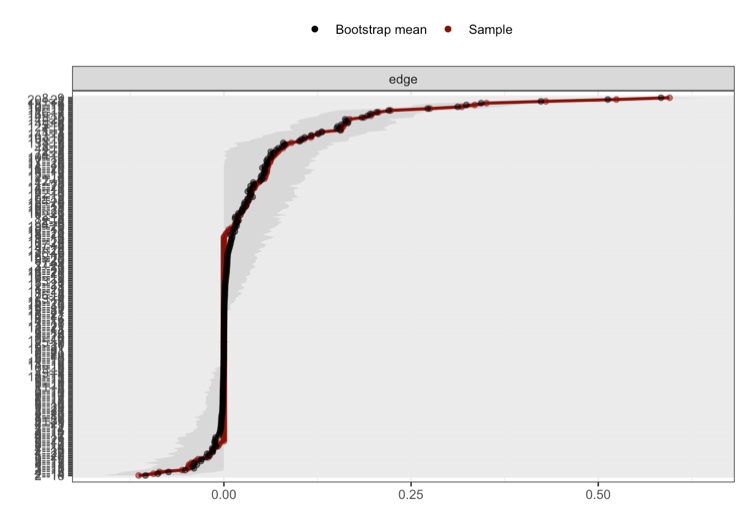
Figure S4. Bootstrapped results of the main network model.**

*Note.* Betweenness: 0.206; Closeness: 0.516; Edge: 0.75; Expected Influence: 0.516; Strength: 0.75.

**Figure S5. Network models for boys (A) and girls (B) subgroups.**


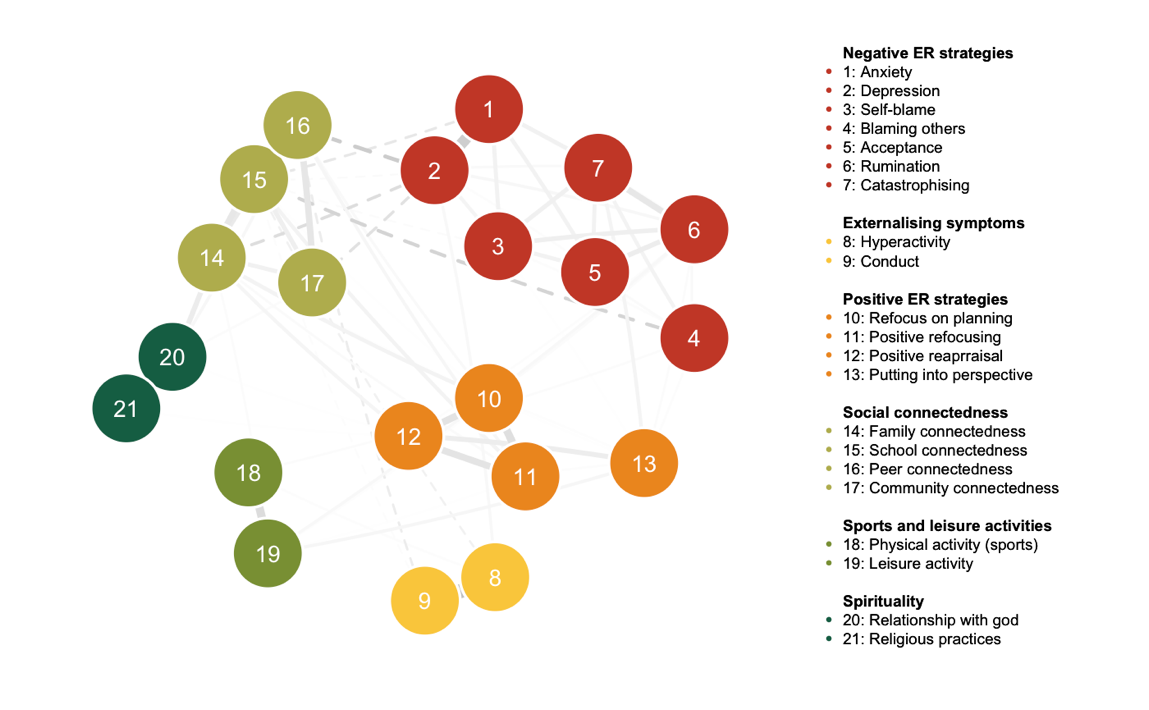


A


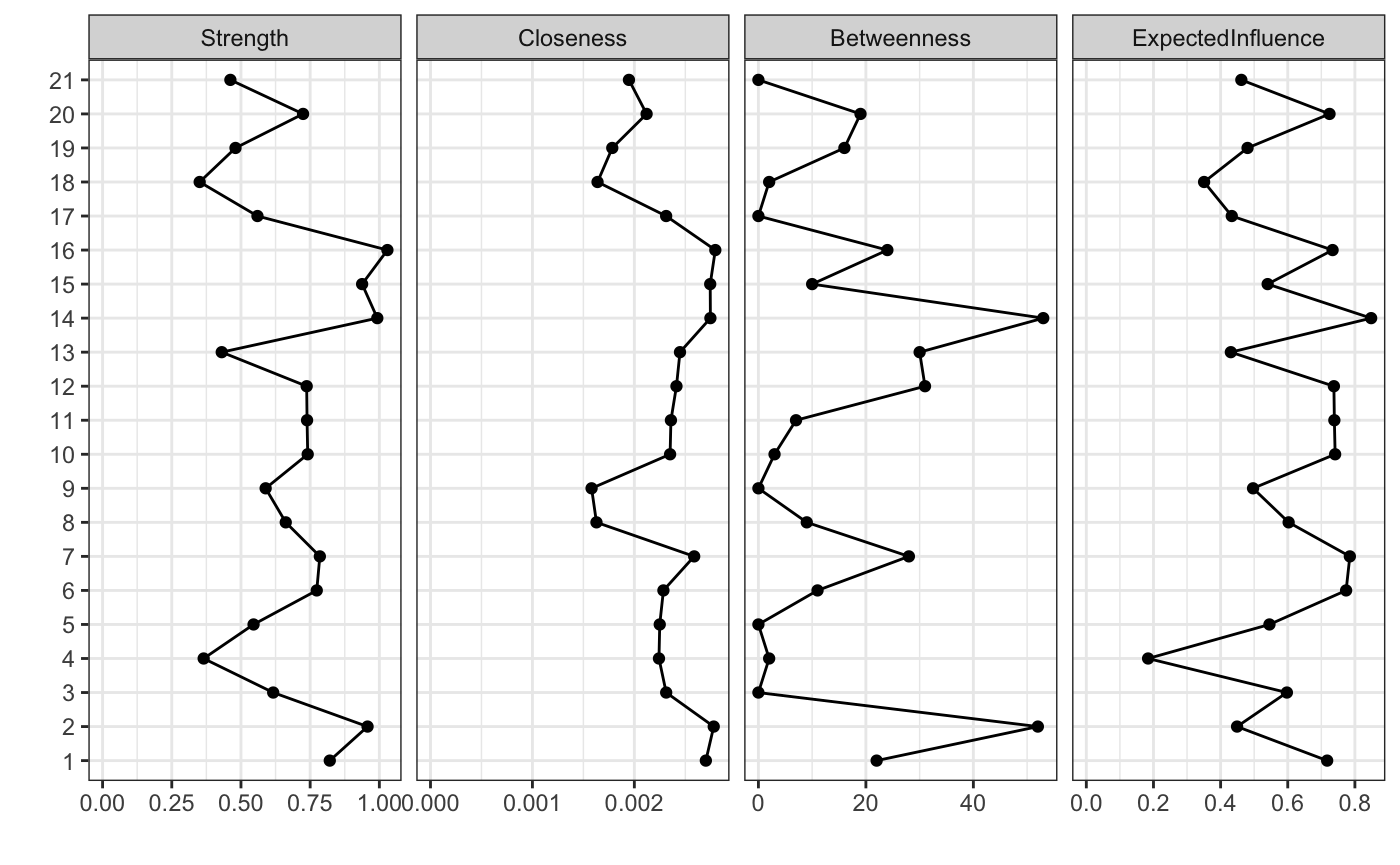


B


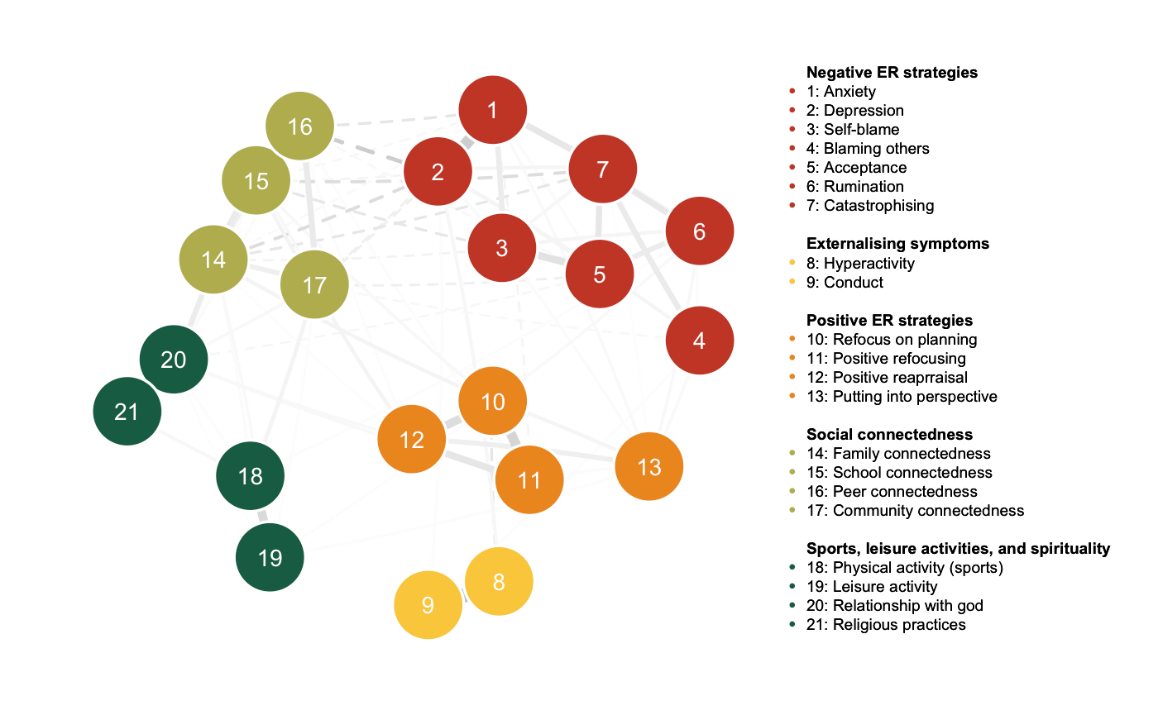

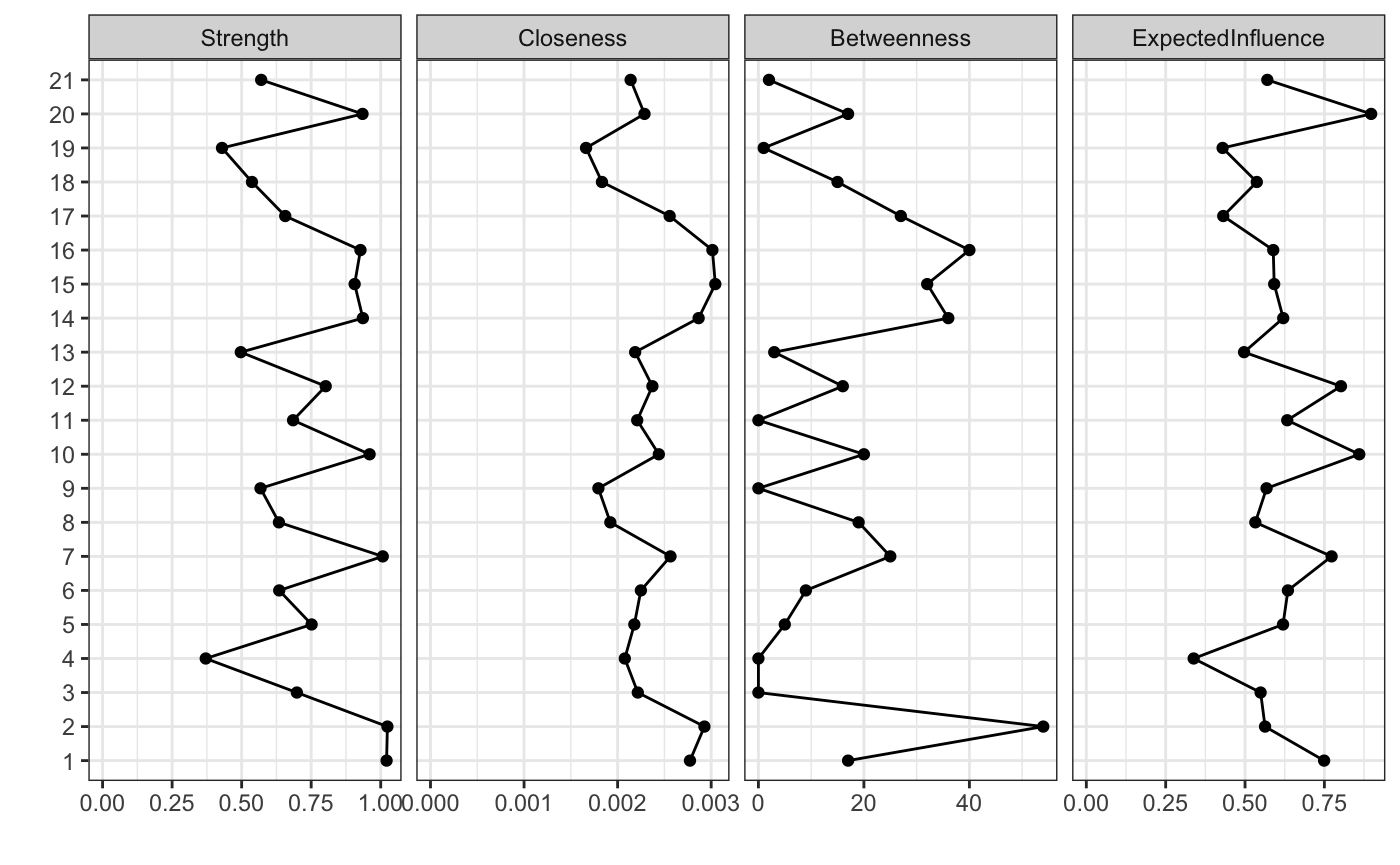


*Note.* Network models and node strength index of resources and mental health for the two gender groups. A) Network model for boys with 6 clusters, B) girls with 5 clusters (note clusters 5 and 6 from the main model are now one).

**Table S1. Demographics based comparisons of resource outcomes.**

| **Measure** | ***M* (*SD*, *n*)** | | **Test statistic*** |
| --- | --- | --- | --- |
|  | ***Sex*** | | |
|  | **Male** | **Female** |  |
| *CERQ-k* |  |  |  |
| Self-blame | 4.17 (1.98, 398) | 4.21 (1.92, 390) | *U*=76113.5, p=0.82 |
| Blaming others | 3.83 (1.94, 398) | 3.56 (1.87, 390) | *U*=84232, p=0.12 |
| Acceptance | 4.69 (2.15, 398) | 4.43 (2.09, 390) | *U*=83325, p=0.19 |
| Rumination | 5.07 (2.15, 398) | 4.87 (2.13, 390) | *U*=82335, p=0.29 |
| Catastrophising | 4.85 (2.25, 398) | 4.49 (2.15, 390) | *U*=84948, p=0.09 |
| Refocus on planning | **7.11 (2.27, 398)** | **6.65 (2.24, 390)** | ***U*=86872, p=0.03** |
| Positive refocussing | 6.94 (2.39, 398) | 6.6 (2.42, 390) | *U*=83920.5, p=0.15 |
| Positive reappraisal | 6.39 (2.3, 398) | 6.06 (2.29, 390) | *U*=84669.5, p=0.10 |
| Putting into perspective | 5.67 (2.31, 398) | 5.45 (2.28, 390) | *U*=81918, p=0.34 |
| *SCQ* |  |  |  |
| Family | 45.28 (7.24, 395) | 45.29 (7.24, 389) | *U*=76614.5, p=0.96 |
| School | **23.38 (5.04, 395)** | **24.54 (4.38, 389)** | ***U*=66843.5, p=0.01** |
| Peer | 28.13 (5.09, 395) | 28.62 (5.1, 389) | *U*=71625.5, p=0.24 |
| Community | 14.46 (3.39, 395) | 14.53 (3.26, 389) | *U*=75859.5, p=0.88 |
| *CLASS* |  |  |  |
| Physical activity | 54.84 (14.82, 358) | 55.81 (14.49, 358) | *U*=60866.5, p=0.40 |
| Leisure activity | 25.41 (5.65, 354) | 25.83 (5.42, 360) | *U*=61950, p=0.72 |
| *YSS* |  |  |  |
| Relationship with God | 3.96 (0.88, 387) | 3.99 (0.82, 379) | *U*=72651.5, p=0.88 |
| Religious practices | 3.41 (1.21, 387) | 3.47 (1.17, 379) | *U*=71301.5, p=0.71 |
|  | ***Free school meals*** | | |
|  | **“No”** | **“Yes”** |  |
| *CERQ-k* |  |  |  |
| Self-blame | 4.25 (1.96, 574) | 4.04 (1.98, 185) | *U*=57031, p=0.27 |
| Blaming others | 3.71 (1.92, 574) | 3.65 (1.85, 185) | *U*=53632, p=0.88 |
| Acceptance | 4.58 (2.16, 574) | 4.5 (2.06, 185) | *U*=53656.5, p=0.88 |
| Rumination | 5.03 (2.18, 574) | 4.82 (2.04, 185) | *U*=55585, p=0.51 |
| Catastrophising | 4.68 (2.17, 574) | 4.62 (2.29, 185) | *U*=54683, p=0.73 |
| Refocus on planning | 6.94 (2.23, 574) | 6.69 (2.35, 185) | *U*=56436.5, p=0.35 |
| Positive refocussing | 6.82 (2.38, 574) | 6.49 (2.46, 185) | *U*=57349, p=0.24 |
| Positive reappraisal | 6.21 (2.28, 574) | 6.18 (2.31, 185) | *U*=53672, p=0.88 |
| Putting into perspective | 5.64 (2.22, 574) | 5.44 (2.45, 185) | *U*=56220, p=0.38 |
| *SCQ* |  |  |  |
| Family | 45.21 (7.21, 570) | 45.08 (7.55, 185) | *U*=52937.5, p=0.96 |
| School | 24.05 (4.69, 570) | 23.5 (4.8, 185) | *U*=56391.5, p=0.31 |
| Peer | 28.61 (4.72, 570) | 27.76 (5.91, 185) | *U* =55284, p=0.51 |
| Community | 14.72 (3.19, 570) | 13.98 (3.62, 185) | *U*=57580.5, p=0.17 |
| *CLASS* |  |  |  |
| Physical activity | 54.74 (13.82, 522) | 57.01 (16.13, 171) | *U* =41602, p=0.34 |
| Leisure activity | 25.71 (5.51, 512) | 25.59 (5.68, 174) | *U* =45060, p=0.88 |
| *YSS* |  |  |  |
| Relationship with God | 3.93 (0.88, 556) | 4.08 (0.8, 181) | *U*=44936.5, p=0.11 |
| Religious practices | 3.39 (1.23, 556) | 3.6 (1.12, 181) | *U*=45838, p=0.19 |

| **Measure** | ***Age*** | | |
| --- | --- | --- | --- |
|  | **Beta (SE)**** | | **p value** |
| *CERQ-k* |  | |  |
| Self-blame | 0.02 (SE=0.07) | | p=0.83 |
| Blaming others | **-0.18 (SE=0.07)** | | **p=0.03** |
| Acceptance | -0.12 (SE=0.08) | | p=0.19 |
| Rumination | -0.1 (SE=0.08) | | p=0.31 |
| Catastrophising | -0.07 (SE=0.08) | | p=0.46 |
| Refocus on planning | 0.16 (SE=0.08) | | p=0.10 |
| Positive refocussing | **0.29 (SE=0.09)** | | **p=0.01** |
| Positive reappraisal | **0.2 (SE=0.09)** | | **p=0.04** |
| Putting into perspective | **0.2 (SE=0.09)** | | **p=0.04** |
| *SCQ* |  | |  |
| Family | **1.18 (SE=0.27)** | | **p<0.01** |
| School | 0.07 (SE=0.18) | | p=0.77 |
| Peer | 0.24 (SE=0.19) | | p=0.28 |
| Community | **0.33 (SE=0.12)** | | **p=0.02** |
| *CLASS* |  | |  |
| Physical activity | -0.09 (SE=0.59) | | p=0.88 |
| Leisure activity | **0.97 (SE=0.22)** | | **p<0.01** |
| *YSS* |  | |  |
| Relationship with God | **0.17 (SE=0.03)** | | **p<0.01** |
| Religious practices | **0.27 (SE=0.04)** | | **p<0.01** |
|  | ***Ethnicity category*** | | |
|  | **F(df)**** | **p value** | |
| *CERQ-k* |  |  | |
| Self-blame | F(4,754)=0.56 | p=0.69 | |
| Blaming others | **F(4,754)=2.94** | **p=0.02** | |
| Acceptance | **F(4,754)=2.47** | **p=0.04** | |
| Rumination | F(4,754)=0.39 | p=0.82 | |
| Catastrophising | F(4,754)=1.56 | p=0.18 | |
| Refocus on planning | F(4,754)=2.02 | p=0.09 | |
| Positive refocussing | F(4,754)=1.95 | p=0.10 | |
| Positive reappraisal | **F(4,754)=3.75** | **p=0.01** | |
| Putting into perspective | F(4,754)=1.66 | p=0.16 | |
| *SCQ* |  |  | |
| Family | F(4,750)=0.52 | p=0.72 | |
| School | F(4,750)=1.07 | p=0.37 | |
| Peer | F(4,750)=0.05 | p=0.99 | |
| Community | F(4,750)=0.64 | p=0.64 | |
| *CLASS* |  |  | |
| Physical activity | F(4,687)=0.95 | p=0.43 | |
| Leisure activity | F(4,682)=0.7 | p=0.59 | |
| *YSS* |  |  | |
| Relationship with God | **F(4,732)=37.06** | **p < 0.01** | |
| Religious practices | **F(4,732)=30.92** | **p < 0.01** | |

*Note.* *Sex and ‘free school meals’ group comparisons conducted with Mann-Whitney U test.

**Age effects on outcomes tested with linear regressions. ***Ethnicity categories compared with ANOVA. All *p* values have been corrected for multiple comparisons with the Benjamini-Hochberg procedure (FDR).
